# Feasibility of a physical activity programme embedded into the daily lives of older adults living in nursing homes: protocol for a randomised controlled pilot feasibility study

**DOI:** 10.1186/s13063-018-2848-4

**Published:** 2018-08-29

**Authors:** Eva Barrett, Paddy Gillespie, John Newell, Dympna Casey

**Affiliations:** 1 School of Nursing and Midwifery, National University of Ireland Galway, Galway, Ireland; 2 Health Economics and Policy Analysis Centre, National University of Ireland Galway, Galway, Ireland; 3 School of Mathematics, Statistics and Applied Mathematics, National University of Ireland Galway, Galway, Ireland

**Keywords:** Physical activity, Nursing home, Feasibility, Older adults, Qualitative, Economic

## Abstract

**Background:**

Older adults living in nursing homes spend the majority of their time inactive. The associated levels of chronic disease place an increasing burden on healthcare systems. Physical activity (PA) interventions delivered through exercise classes may be resource-intensive and require specialist staff. The aim of this study is to explore the feasibility and acceptability of a PA programme embedded into the daily lives of older adults living in nursing homes and to examine the preliminary effects of this on physical mobility and quality of life.

**Methods:**

A randomised controlled pilot feasibility study, including embedded qualitative and economic components will be carried out. Two randomly selected nursing homes will take part in the study; participants (*n* = 20) in one nursing home will receive a three-month PA intervention and participants (n = 20) in the other will be a usual care control. Nursing home staff will be provided with training and support to monitor participants PA programmes. Feasibility data will be collected on recruitment, randomisation, assessment and intervention procedures. Criteria for progression of the pilot feasibility study to a definitive trial will be specified. The Timed Up and Go test, Nottingham Health Profile, 10-Metre Walk test, the Investigating Choice Experiments for the Preferences of Older People Capability index and the Bangor Goal Setting Interview will be assessed at baseline, three-month and 12-month follow-up. Between-group and within-individual effects will be estimated using appropriate linear mixed models. Semi-structured interviews will be conducted with staff and participants of the intervention group within one month after the intervention to explore the feasibility and acceptability of the programme. A subset of control participants will be interviewed to describe usual care. Economic data will be collected to examine costs of the intervention in comparison with costs in the control group.

**Discussion:**

The findings will facilitate refinement of the PA programme and development of a clear protocol for subsequent evaluation of the PA intervention in a definitive randomised controlled trial.

**Trial registration:**

ClinicalTrials.gov, NCT03484715. Registered on 30 March 2018.

**Electronic supplementary material:**

The online version of this article (10.1186/s13063-018-2848-4) contains supplementary material, which is available to authorized users.

## Background

Globally, the number of older adults unable to live independently is forecast to quadruple by 2050 [1]. The rapid change in population demographics will make nursing homes a vital pillar of healthcare for future older generations. Physical inactivity is a global health pandemic and is one of four leading contributors to premature mortality [2]. In nursing homes, the consequences of physical inactivity are even more apparent and may manifest as pressure sores, contractures, cardiovascular deconditioning, urinary infections and increased dependency [3]. The World Health Organization (WHO) guidelines advocate that older adults undergo 150 min of physical activity (PA) each week, performed in at least 10-min bouts [4]. Despite this, people living in nursing homes spend the majority of their time inactive [5]. A large, observational study conducted in The Netherlands reported that nursing home residents can spend 89–92% of their waking hours in sitting or lying positions [6].

A recent Cochrane review concluded that PA improves short-term function and is safe among older adults of long-term care [7]. However, the optimum contents of a PA programme or how best to implement it remain unknown [7]. Previous research has typically delivered PA programmes through exercise classes [8, 9]. While demonstrating small improvements in health outcomes, this approach can be time-consuming and resource-intensive, requiring extra equipment and specialist staff [10, 11]. As such, the sustainability of PA programmes in nursing homes has been highlighted as a particular challenge [7]. Randomised controlled trials (RCTs) assessing feasible ways to increase PA levels in nursing homes are needed, especially if long-term uptake is intended. Furthermore, to date there is limited economic evidence supporting PA programmes in nursing homes, which necessitates more cost-effectiveness evaluations to be included in trials [7].

Few studies in nursing homes have attempted to increase PA levels by embedding additional functional activities into the resident’s daily routine [12]. A person-centred programme of progressive repetitions of activities of daily living has been shown to increase the ability of older adults to perform these activities independently [13], which may provide a means of encouraging regular physical activity. Furthermore, ensuring that an intervention is perceived as relevant and meaningful by participants may be vital to its success [14, 15]. Incorporating the PA programme into the resident’s everyday routine may help to portray the relevancy of the PA programme to their lives, promoting increased participation and creating a sustainable means of increasing PA levels in the long term.

A further challenge is that older aged participants are commonly reported as having poor compliance with PA interventions [12, 16], for reasons including poor health status, poor outcome expectations, low self-efficacy, lack of support and environmental barriers [17]. Social cognitive theory (SCT) provides a framework to support participants in making positive behaviour changes [18]. This theoretical framework consists of a number of principles including: *knowledge* of health benefits arising from engaging in the positive behaviour change; a person’s *self-efficacy* that they can achieve the desired outcomes; *outcome expectations* about the expected costs and benefits of engaging in the given behaviour; personal *goals* which guide the path to behaviour change; and *perceived impediments and facilitators* to engaging in the desired behaviour change. Incorporating these constructs into PA interventions has been found to be effective in generating positive behaviour changes toward PA in the past [19, 20].

The proposed pilot feasibility study will implement a person-centred PA programme into the participant’s daily routine, based on increasing duration and repetitions of everyday functions including walking and rising from a chair. It incorporates key constructs of SCT and is monitored by nursing home staff after an initial training and support period.

### Purpose of the study

The primary aim of this pilot feasibility study is to assess the feasibility and acceptability of a PA intervention embedded into the daily lives of older adults living in nursing homes. The secondary aim is to examine the preliminary effects of this on physical mobility and quality of life, compared to participants receiving usual care. It is intended that this will facilitate the refinement of the PA programme where needed and will facilitate the development of a clear protocol for subsequent evaluation of the PA intervention in a larger, definitive RCT. The primary and secondary research questions are listed in Table 1.

**Table 1:** Primary and secondary research questions

**Primary research questions**	
Is the intervention feasible and acceptable to staff and participants? • Is the time commitment required for staff to monitor the intervention and participants to partake in the intervention feasible? • Are there any environmental barriers to completion of the intervention? • Is the content of the intervention acceptable to participants and staff? • Do the participants adhere to the intervention and what adherence issues arise? • What are the components of usual care for participants allocated to the control group?	
Are the outcome measures feasible and acceptable to participants? • What is the required time and number of visits required to collect each outcome from participants? • Are the outcome measures acceptable to participants? • What is the level of missing data within the self-reported outcome measures? • What is the feasibility of collecting health economic data (i.e. sources used and time taken)?	
What is the required sample size for a definitive RCT? • What is the baseline score and variability of the primary outcome measure among participants? • What is the estimated effect size and variance for the primary outcome measure? • What are the recruitment and attrition rates for nursing homes and participants? Do these rates differ between the intervention and control groups?	
**Secondary research questions**	
What are the preliminary clinical outcomes and cost-effectiveness of the intervention compared to the control group? • What are the estimated outcomes of the intervention in comparison with the control group and are these sustained at 12-month follow-up? • What are the costs of the intervention in comparison to costs in the control group?	

## Methods

### Design and setting

**Fig. 1:**
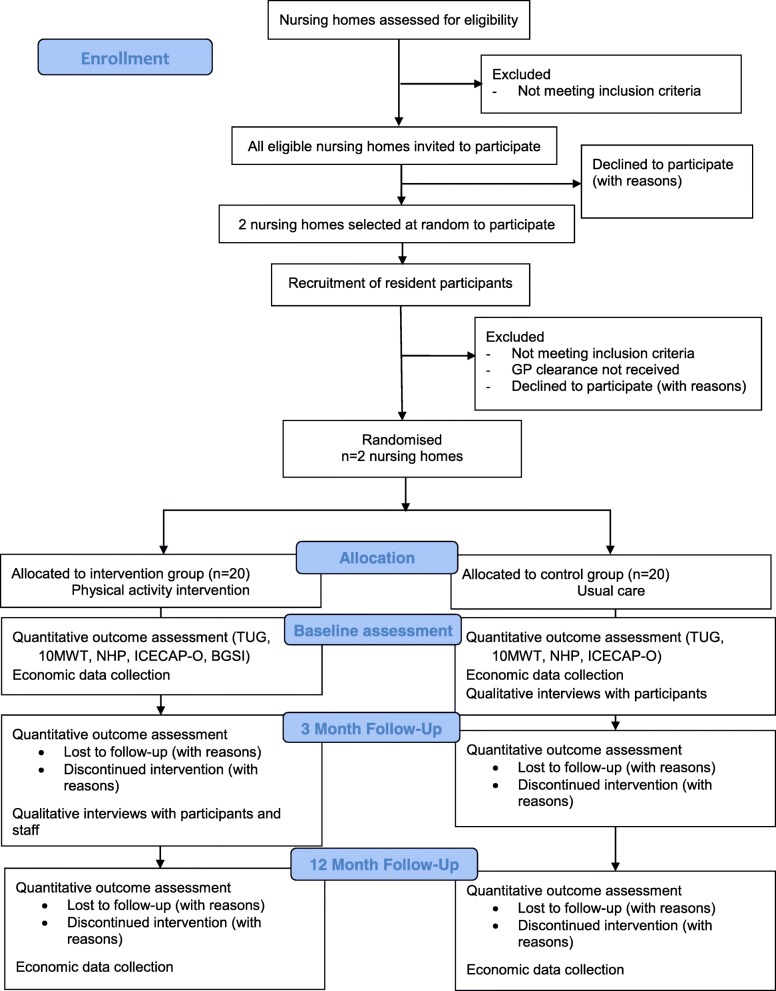
CONSORT flow diagram

**Fig. 2:**
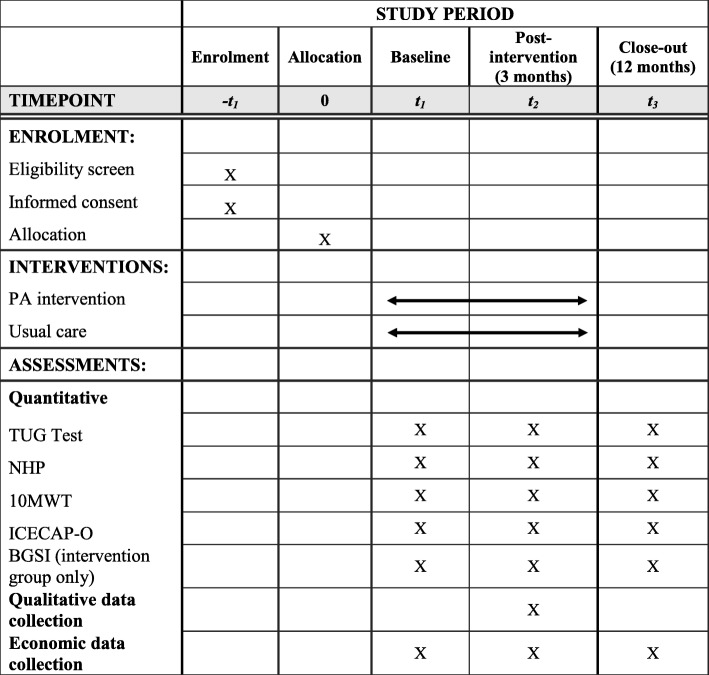
*Schedule* of enrolment, interventions and assessments as per SPIRIT 2013

A randomised controlled pilot feasibility study, with embedded qualitative and economic components, will be carried out. Two nursing homes will take part in the study; participants in one nursing home will receive the PA intervention and participants in the other will receive usual care. In order to avoid contamination between intervention and control groups, randomisation will take place at nursing home level [12]. The pilot feasibility study will not be an exact scale model of a definitive RCT, the difference being that follow-up outcome assessment will not be blinded as a full analysis of outcome data is not intended. However, the recruitment, randomisation, assessment and intervention conditions will be similar to a definitive RCT in order to answer the feasibility research questions. Figure 1 provides a CONSORT flow diagram displaying the movement of participants throughout the study. A schedule for enrolment, interventions and assessments is displayed in Fig. 2. The protocol adheres to the Standard Protocol Items Recommendations for Interventional Trials (SPIRIT) guidelines (Additional file 1) [21] and the SPIRIT-Patient Reported Outcomes Extension [22].

### Sites and participants

The pilot feasibility study will take place in two Irish nursing homes. Nursing home and participant eligibility criteria are outlined in Table 2.

**Table 2:** Eligibility criteria for nursing homes and participants

Inclusion criteria	Exclusion criteria
Nursing homes	
• Registered nursing homes, either public or private. In a definitive trial, an equal number of private and public nursing homes should be allocated to each arm, in order to provide similar conditions for comparison. As this pilot feasibility study will have one nursing home in each arm, both nursing homes in the current study must be either public or private. • Located within a 30-km radius from National University of Ireland Galway (to allow the research team to provide ongoing site visits and support). • Minimum capacity of 45 residents in the nursing home (to ensure sufficient participant recruitment based on previous research [54]). • Willingness and agreement of managers to assist with the study protocol where required, including releasing relevant staff where needed and identifying residents eligible for participation.	• Voluntary nursing homes, retirement centre, day care centre, home care services or hospital. • Currently taking part in another research study which influences PA behaviour of resident. • Does not satisfy all of the above inclusion criteria.
Participants	
• Aged 65 years or over. • Can speak and understand English. • Resident in care home for at least 3 months (to allow the resident an appropriate adjustment period and time to establish daily routine). • Able to rise from a chair with armrests (with or without a walking aid) and able to walk at least 10 m (with or without a walking aid) independently or with minimal assistance. • Approval of the resident’s GP to participate (to ensure participant safety). • In the professional judgement of the key nurse caring for the resident, the resident must have the cognitive capacity to engage in the goal-setting and PA aspects of the intervention and be likely to benefit from taking part in the study. • Informed consent is obtained for participation through either (i) resident providing written informed consent or (ii) resident providing assent to participate and written informed consent obtained from the resident’s next of kin.	• Admission to nursing home for respite or end-stage terminal care. • A significant sensory impairment, physical impairment or illness that impairs their ability to participate. • Uncontrolled cardiovascular, musculoskeletal or neurological disorders.

### Sample size calculation

An objective of this study is to assess the feasibility of recruiting and retaining the required number of participants per nursing home which would be needed in a definitive RCT. Therefore, the sample size calculation for this pilot feasibility study is based on the estimated power calculation for a definitive cluster RCT using the primary outcome measure, the Timed Up and Go (TUG) test [23]. In the absence of normative TUG data for an Irish cohort of older adults living in nursing homes, sample size calculations for a definitive RCT were estimated using a New Zealand sample of older adults living in residential care [12]. The sample size requirements for a definitive RCT were calculated as being a minimum of four clusters of size 20 in each arm, in order to have 80% power (α = 0.05) to detect a mean change in TUG score of 4.7 s (± 6.8 s). The current study will recruit one nursing home for each arm. The desired number of participants in each nursing home will be maintained as 20 in order to assess the recruitment potential, recruitment time and feasibility of delivering the intervention to this number of participants. The number of participants lost to follow-up per nursing home will be recorded to inform the final numbers needed when recruiting to the definitive RCT.

### Recruitment

#### Recruitment of nursing homes

The Directory of Nursing Homes [24] is a comprehensive listing of all public and private nursing homes in Ireland and will be used to identify all potentially eligible nursing homes in County Galway. The reason for non-eligibility of nursing homes from this list will be documented. All eligible nursing homes will be posted an invitation letter along with a booklet containing information about the study. This will be promptly followed up with a telephone call, where the researcher will attempt to speak directly with the Director of Nursing (DON) or another person in charge. The nursing home will be provided with two weeks to consider participation. At this point, if the nursing homes would like to further explore participation in the study, a meeting will be convened between the researcher and DON. The equal likelihood of being in the intervention or control arm of the study will be clearly explained to all nursing homes from the outset. A list of nursing homes willing to participate will then be created. From this list, one nursing home will be randomly selected, by a statistician independent of this study, to receive the intervention and a second will be randomly chosen for the control. The group allocation will be concealed from potential participants, the nursing home and the researchers involved in recruitment and assessment in a sealed opaque envelope until all participants have been recruited to the study and have completed baseline assessment.

#### Recruitment of participants

The DON in each nursing home or her nominee will identify all residents who satisfy the eligibility criteria. The main reasons for residents not meeting these eligibility criteria will be documented, as this will provide useful feasibility data. General practitioner (GP) clearance will be sought for each potential participant before they are invited to participate. Once this screening is complete, a member of staff will ask potential participants if they would be willing to talk to a researcher about participating in a PA research study. Once a potential participant agrees, the staff member will introduce the researcher to the potential participant. The researcher will explain the study in simple terms and explore whether the resident is interested in participating. In the case of the resident expressing a disinterest in the study, no further actions will be pursued and the reason for their lack of interest will be documented. If interested, the resident will be provided with an information leaflet. This document will be reviewed verbally with the participant in order to overcome any potential literacy difficulties and large font sizes will be used. The information sheet will clearly inform participants of the purpose, process, potential benefits and harms of the study, data collection procedures, time commitment, its voluntary nature, as well as providing an assurance of confidentiality. The equal likelihood of receiving the intervention or control arm of the study will be clearly explained to all participants, as well as their freedom to withdraw from the study at any stage without any consequence to their care.

Where a participant expresses an understanding of the study, its voluntary nature and a choice to participate, the researcher will finalise the consent process directly with the person. In instances where it is not possible for the potential participant to provide written informed consent, assent will be obtained from the participant. In line with current evidence [25], assent will be obtained from the participant by an affirmative agreement to participate as expressed verbally (i.e. orally) or a non-verbal indication of willingness to cooperate with study procedures, i.e. behaviourally (e.g. acting agreeably) or emotionally (e.g. having a positive facial expression). In addition to assent, the person’s next of kin will be asked to provide written informed consent on behalf of their relative. The next of kin will be asked to make their decision on the basis of their knowledge of the person’s wishes, attitudes and values. Assent will be assessed continually throughout the study for all participants.

### Intervention

Key elements of SCT have been incorporated into the PA intervention, using the strategies outlined in Table 3. The PA intervention will comprise three components led by a researcher who is a qualified physiotherapist.

- Participant goal-setting

**Table 3:** Specific intervention strategies and how they relate to each theoretical construct [18]

Behaviour change principle	Intervention strategies
Knowledge	- Researcher will educate each participant on health benefits of PA in the context of their own health - Educational posters displayed in nursing home - Re-enforcement by the care staff monitoring the intervention
Self-efficacy	- Completion of baseline physical assessments - Involvement of participants in refining their personal PA programme - Mastery of PA programme throughout intervention - Observation of other participants being physically active - Encouragement and constructive feedback from researcher and staff
Outcome expectations	- Discussion of outcome expectations in initial meeting between researcher and participant - Self-monitoring of PA programme via logbook - Reassessment of goals at follow-up to chart improvement
Goal-setting	- Participant sets PA related goals and performance indicators - Goals and PA programme displayed on bedroom wall - Reassessment of goal attainment at follow-up
Perceived impediments and facilitators	- Barriers and facilitators to goal attainment discussed and addressed in initial meeting - Allocation of staff to monitor intervention and support participants with their PA programmes - Reflection on personal diary - Participants encouraged to carry out walking together for social support

The researcher will meet with each participant individually to educate them of the benefits of PA in their own context and to facilitate participants in setting a small number of activity-based goals that they would like to achieve over the intervention period. Goals should be in relation to performing a physical task, such as walking to the dining area for meals, participating in particular group activities or gardening. The Bangor Goal Setting Interview [26] will provide a structured format for the goal-setting process. First, the researcher will facilitate the participant to explore different domains of daily life and identify issues of importance to them that may form the basis of goals. The importance for change and readiness to change in each of these areas will be explored using the principles of motivational interviewing. The researcher will facilitate the participant in revisiting each area and choosing the desired goals using SMART (specific, measurable, achievable, realistic and timely) criteria. The researcher and participant will work together to set goal attainment indicators, which will provide clear descriptors of what would constitute 25%, 50% and 75% goal attainment [26]. Finally, the participant will be asked to rate their current performance and satisfaction with performance on a Likert scale of 1–10 (where 1 means cannot do or am not doing successfully and 10 means can do and am doing very successfully). A visual representation of this scale will be used to assist participants. A separate, independent rating of goal attainment will be recorded by the researcher following observation. Barriers and facilitators to achieving the goal will be discussed with an emphasis on resources that will help in overcoming obstacles and achieving their goals.

b) PA programme

Using the information gathered from the baseline assessment and goal-setting exercise, a PA programme will be developed by the researcher and tailored to each participant. The participant will be given an opportunity to refine the PA programme with the researcher before it is finalised, as participant satisfaction with PA interventions is important for adherence [27]. PA programmes will be carried out on three days per week. Integral to each PA programme will be a walking component (i.e. structured and gradual increases in daily walking time) and sit-to-stand exercises (i.e. a specified number of repeated rises from a chair). These are functional and accessible means of providing the aerobic and resistance components considered as important to PA programmes [28]. Opportunities for walking will be identified with each participant and the care staff. This will be based on lengthening walks that the participant is already undergoing. For example, the participants’ walk to or from the dining area or their walk from the bedroom to the seating area will be increased by 1–5 min initially and progressed to a maximum of 10 min. Where possible, participants will be encouraged to walk with others, as exercising with peers has been shown to enhance adherence to and persistence with an exercise program among older adults [29]. Sit-to-stand exercises will be embedded into the participant’s daily routines, for example rather than rising from a chair once, participants will complete a series of sit-to-stands in their bedroom, the dining room or the seating area. Depending on individual ability, this will begin as two sets of 2–5 repetitions initially and progressed to a maximum of three sets of 15 repetitions. The remaining components of the PA programme will be tailored around each participant’s specific functional goals and will be based on repeating particular functional and meaningful activities. The researcher will progress each PA programme as tolerated by each participant. All participants in the intervention group will commence the PA programme at the same time.

A poster displaying the participant’s goals and PA programme will be visible on the wall in their bedroom to promote adherence. Where possible, participants will be encouraged to document their own adherence to their PA programme using an easy-to-complete diary provided by the researcher, detailing the time spent walking and the number of repetitions achieved for each activity. Where cognitive or physical barriers prevent participants from completing their own adherence diary, the staff will be responsible for completing the diary for them. When the PA occurs upon the participant getting up in the morning, the staff member assisting the participant in getting up will be responsible for completing the adherence diary. For daytime PA, the staff member who supervises the PA will document adherence on a centrally placed progress board. Space will be provided to detail any deviations or modifications of the PA programme. Recording adherence to PA on a daily basis like this provides the most detailed data [30] and is less susceptible to recall errors which are evident in many retrospective PA questionnaires [31].

c) Staff training and support

All nursing home staff will be invited to attend two education sessions. The first session will take place at the beginning of the intervention and the second at the midway point. Table 4 outlines the content of these educational sessions. Staff will be responsible for overseeing the completion of the participant’s PA programmes which may involve providing reminders, encouragement or assistance with PA programmes and assistance with documenting adherence. The researcher will support the nursing home staff to supervise the participants PA programmes through twice weekly support visits and ongoing telephone support. Posters will be displayed on the nursing home notice boards to highlight the impact of PA on health and wellbeing and to generate awareness of the study among nursing home staff, residents and visitors.

**Table 4:** Content of two staff education sessions

Session 1 (2 h)	
(i) Welcome, introduction and the importance of PA for older adults	
(ii) What the intervention aims to achieve and the rationale for the intervention	
(iii) Overview of the goals set by participants	
(iv) Implementation plan, including the structure on supervising participants with their programmes, completing the adherence sheets and addressing potential barriers to implementation	
(v) Techniques for educating participants on the benefits of PA and motivating participants with their programmes	
(vi) Questions and answers	
Session 2 (1 h)	
(i) Evaluation on progress of the study	
(ii) Addressing issues which may be challenging the implementation of the programme	
(iii) Methods to assist participants’ continued engagement with their programme	

### Control group

The participants in the control group will receive usual care, which will be guided by current nursing and medical care plans. Participants in the control group are not restricted in any way regarding their PA behaviour. However, participants will be asked to document any changes in PA which occurs during the intervention period and the researcher will verify this with the key nurse at each follow-up. The feasibility and time taken to collect this information will be documented. At the end of the intervention, if a positive outcome is indicated, the nursing home staff which takes part in the control arm will be provided with the materials used in the intervention arm. The researcher will also deliver an educational session and guidance on how to deliver the programme in their nursing home.

### Data collection

#### Descriptive measures

Demographical information, including age, gender, length of time in nursing home, Mini Mental State Examination (MMSE) score, co-morbidities, medications, use of walking aid and current PA habits, will be collected to describe both groups at baseline.

#### Feasibility data

The feasibility of the study processes are key outcomes of this study. During the recruitment process, the proportion of nursing homes which satisfy the inclusion criteria, reasons for ineligibility of remaining nursing homes and the proportion of invited nursing homes that agree to participate will be recorded. Similarly, for participant recruitment, the proportion of residents in each care home who are eligible and who accept to participate will be documented, as well as reasons for ineligibility and lack of interest. This will elucidate if the eligibility criteria for nursing homes and participants are appropriate or too restrictive. The time taken for recruitment of nursing homes and participants will be documented. Attrition rates of the nursing homes and participants at each time point will be documented. The length of time and number of visits required to collect each outcome from participants will be documented, as well as the proportion of missing data. The components of the study which are critical to enabling a future definitive RCT will be assessed using the progression criteria outlined in Table 5. The cut-off values for each criterion are based on average rates reported by studies in a previous systematic review [7]. A traffic light system of green (continue without modifications – feasible as is), amber (continue, but modify protocol – feasible with modifications) and red (stop – main study not feasible) will be used, as advised by the Medical Research Council Hubs for Trials Methodology Research [32].

**Table 5:** Criteria for progression of pilot feasibility study to a definitive trial

Feasibility outcome	Progression criterion
Recruitment	
1. Proportion of eligible nursing homes which agree to participate in study	≥ 70% of eligible nursing homes agree to take part in the study, proceed; 30–70% agree to take part, modify protocol; < 30% agree to participate, do not proceed.
2. Adequate participant recruitment rate	Recruitment rate in each nursing homes of > 80% of required (*n* = 16), proceed; 60–80% of required (*n* = 12–15), modify protocol; < 60% of required (*n* < 12), do not proceed.
3. Average time for recruitment of nursing homes and participants	Nursing homes Measured from initial contact to decision to participate: If < 4 weeks, proceed; if 4–8 weeks, modify protocol; if > 8 weeks, do not proceed Participants Measured from initial contact to consent obtained: If < 3 weeks, proceed; if 3–5 weeks, modify protocol; if > 5 weeks, do not proceed
Outcome assessment	
4. Adequate retention rate of nursing homes and participants at 3-month and 12-month follow-up	Nursing homes Both nursing homes are required to complete the 12-month follow-up; Participants Measured using the following scale in both the intervention and control group: At 3 months: if > 80% retained, proceed; if 60%–80% retained, modify protocol; if < 60% retained, do not proceed At 12 months: if > 70% retained, proceed; if 40%–70% retained, modify protocol; if < 40% retained, do not proceed
Protocol adherence	
5. Adequate adherence rates of participants to the intervention	> 80% of the participants in the intervention arm complete 75% of their physical activity programme, proceed; 60–80% of participants complete 75% of the physical activity programme, modify protocol; < 60% of participants complete 75% of physical activity programme.

#### Outcome measurements

Quantitative outcome data will be collected in an unblinded manner from participants at T1 (baseline, pre-intervention), T2 (directly after completion of the intervention at three months) to examine the immediate intervention outcomes and T3 (12 months following the start of the intervention) to examine if the outcomes are sustained beyond intervention completion.

##### Primary outcome measure

The primary outcome measure will be physical mobility using the Timed Up and Go (TUG) test [23]. This measures the time taken by an individual to stand up from a standard arm chair, walk a distance of 3 m at a comfortable pace, turn, walk back to the chair and sit down. This test has been shown to predict morbidity and mortality in different populations [33, 34] and is recommended by the American Geriatrics Society and British Geriatrics Society as an indicator of function, mobility and falls risk. The time to complete the task is measured in seconds with a stopwatch. Timing commences on the command ‘go’ and stops when the participant is back sitting down. The TUG will be conducted twice and the average of both attempts will be taken. Participants are permitted to use routine walking aids. Inter-rater and intra-rater reliability is very high for this test among community-dwelling older adults [35, 36] and it is sensitive to change [37].

##### Secondary outcome measures

- The Nottingham Health Profile [38] will be used to measure health-related quality of life. This questionnaire measures self-perceived health across six domains: (i) sleep; (ii) mobility; (iii) energy; (iv) pain; (v) emotional reactions; and (vi) social isolation. It has been shown to be reliable, valid [39] and acceptable to older adults, including those with cognitive impairment [40]. The ‘yes/no’ response format makes it easier to administer, than multiple choice Likert scales of other questionnaires. This questionnaire will be administered by the researcher as a structured interview with the participant, as completion rates of questionnaires in older adults tend to improve when the questionnaire is interviewer-administered [41].
- Gait speed will be assessed using the 10-Metre Walk test. Gait speed is an important objective measure of functional mobility, particularly for older adults [42] and is related to various health outcomes, including functional decline and mortality [43]. The participant will walk 10 m and the time is measured for the intermediate 6 m to allow for acceleration and deceleration. The timer is started when the toes of the leading foot cross the 2-m mark and stopped when the toes of the leading foot cross the 8-m mark. This measure has good inter-rater and intra-rater reliability [42, 44].
- The Investigating Choice Experiments for the Preferences of Older People Capability index (ICECAP-O) [45] assesses quality of life more broadly than health-related quality of life. It will provide a measure of capability across the dimensions of attachment (love and friendship), security (thinking about the future without concern), role (doing things that make you feel valued), enjoyment (enjoyment and pleasure) and control (independence). Each dimension has four response options that are described as statements representing: none; a little; a lot; and full capability. It has shown good validity for use in older adults living in nursing homes [46] and those who have mild to moderate cognitive impairment [46, 47].
- The degree of goal attainment as per the Bangor Goal Setting Interview will be reassessed at each follow-up assessment in the intervention group only. The participant will be assisted in re-rating their current performance and satisfaction with performance for their activity-based SMART goal. The researcher will also assess the extent of progress toward achieving the goal using the previously specified goal attainment indicators.

#### Adverse events

It is not anticipated that participants will be at any risk of harm. While falls are a frequent occurrence in nursing homes, safeguards will be in place to reduce the risk of falls and other adverse events (AE), for example, the prerequisite of GP clearance to participate and an individualised activity programme based on physiotherapy assessment. AEs which do occur will be documented by staff on an Adverse Event Reporting Form. The staff will follow the existing policy within the care home relating to the management of a fall or other AE. The researcher will examine AEs on each support visit and make alterations to the PA programme where appropriate.

### Health economic analysis

A preliminary health economic assessment of the intervention relative to the usual care control will be conducted using a cost-effectiveness analysis and cost-utility analysis. The evaluation will be undertaken in a manner consistent with the guidelines issued by Health Information and Quality Authority for the assessment of healthcare interventions and technologies in Ireland. Resource use associated with delivery of the PA intervention will be measured and costed. In particular, resources relating to educational sessions, healthcare professional time input, consumables and materials, equipment and overheads will be measured. A form detailing the resources used by participants over the previous three months, including health service usage, medication usage and private healthcare or study expenses will be completed in both intervention and control groups at baseline, three-month and 12-month follow-up. The researcher will gather this information in consultation with the lead nurse involved in the study, the participant and the participant’s medical notes. The time taken and sources used to obtain this information will be recorded by the researcher. Unit costs will be applied to convert data on resource use to resource costs and total cost variables will be calculated. Data collected from the primary outcome measure, the TUG, at each time point will be used alongside resource usage to provide the basis for the cost-effectiveness analysis. For the cost-utility analysis, effectiveness will be evaluated based on the quality-adjusted life year, which is the preferred outcome measure for economic evaluation and which will be estimated using the EuroQol EQ-5D-5 L instrument [48], assessed baseline, three-month and 12-month follow-up. An incremental cost-effectiveness analysis will be undertaken to provide information on the marginal costs and marginal effects of the intervention relative to the control. The output from the pilot study will provide information on the potential costs and cost-effectiveness of the intervention, as well as the feasibility of collecting the required data, which will inform the design of a definitive RCT.

### Embedded qualitative component

A qualitative descriptive approach [49] will be taken for the qualitative component of the study. A sample of ten participants from the intervention group will be invited to take part in an audio-recorded, one-to-one semi-structured interview within one month of intervention completion. An interview guide will be developed using established guidance [50, 51] and will explore the participant’s experiences of receiving the intervention, the feasibility and acceptability of the intervention, and the perceived effects of the intervention, including potential AEs. The researcher will explore the participant’s adherence to the programme and any adaptations made to it, as well as the acceptability of the recruitment, consent and outcome assessment processes of the study. The interview will also examine the factors which could facilitate or hinder participants in maintaining their PA levels. These could include environmental factors, personal factors or support from care staff.

Staff from the intervention site will also be invited to take part in a one-to-one semi-structured interview, within one month of intervention completion. A variety of staff (manager, nurse, care staff) will be sought in order to maximise variation in the sample. Staff will be included if they have had a role in recruitment or delivery of the intervention. The interview will explore the staffs’ role and experiences of being involved in the study, including their participation in the staff education sessions and the monitoring of the PA programmes. It will also explore the staff’s perceptions of the potential for sustaining the PA programme within the nursing home following the study.

Semi-structured qualitative interviews will be conducted with a small subset of participants from the control group. It will focus on obtaining a description of usual care and evaluating participant’s experiences during recruitment.

### Statistical analysis

#### Quantitative analysis

The data analysis software SPSS Statistics (24.0 for Windows, Chicago, IL, USA) will be used to manage and analyse quantitative data. Feasibility data will be collated using descriptive statistics and, where relevant, evaluated against progression criteria. Demographical information in both groups will be tabulated and summarised. The effect of the intervention on clinical outcomes will be examined using suitable numerical and graphical summaries. The statistical analysis will serve primarily to provide an estimate of the likely effect size and variability of the response within an individual over time. This, as well as the mean and standard deviation of the primary outcome measure at baseline and the participant attrition rate, will enable a more accurate sample size calculation for a definitive RCT. All within- and between-group outcome analyses will be treated as preliminary and interpreted with caution.

#### Qualitative analysis

Participant and staff interviews will be transcribed verbatim. NVivo qualitative data analysis software (QSR International Pty Ltd., Version 11, 2015) will be used to assist in qualitative data analysis and management. The data will be analysed thematically, guided by the six-stage framework outlined by Braun and Clarke [52]. The data will be initially coded according to the participants own words and these will be sorted into preliminary broad themes. These will then be revised and refined to ensure that there is sufficient distinction between themes and coherence within themes [52].

## Discussion

A large proportion of previous research has implemented PA interventions through an exercise class delivered by exercise specialists and containing specific types of exercises (e.g. muscle strengthening, range of movement, flexibility and balance) [7, 9, 10]. This approach is restrictive for residents as it confines PA to within designated sessions and limits uptake as nursing home staff generally do not have the same expertise as exercise specialists in delivering and monitoring these specific types of exercises. The current protocol will evaluate the feasibility and acceptability of a functionally based, personalised PA intervention embedded into the daily routines of older adults living in nursing homes. If ultimately successful in a large-scale RCT, this may provide an effective and cost-effective means of integrating resident PA into routine clinical practice within the nursing home setting.

The strengths of this study include its clear research questions and provision of criteria for progression to a full-scale RCT, as well as the collection of quantitative, qualitative and economic data in order to gain a comprehensive overview of the intervention. This study will include people with cognitive impairment including people with dementia, thereby increasing the generalisability of the study, as a large proportion of nursing home residents have some level of cognitive impairment [53]. Importantly, this also ensures that people with cognitive impairment are not excluded from research which may potentially benefit their health.

A limitation of the study is the lack of assessor blinding to groups during follow-up outcome assessment. This approach was chosen as the primary aim of the study is to evaluate feasibility and acceptability, rather than to establish concrete effectiveness data. As such, this approach will enable the researcher to gain practical experience of applying the outcomes in this population and to uncover any practical issues which may arise. The preliminary outcome data should be therefore interpreted with care. This study, albeit a pilot feasibility study, is also limited by the small number of nursing homes involved. The subjective means of measuring adherence to the intervention using an exercise diary, as opposed to more objective measures (e.g. video cameras), is a further limitation to the study.

Research involving older adults is important and necessary to better enable the effective management of the ageing population. The findings of this study will be disseminated in peer-reviewed journals and presented at national and international conferences.

### Trial status

Protocol version 6. Recruitment began in March 2018 and will end in June 2018.

## Additional file

Additional file 1: SPIRIT 2013 Checklist: Recommended items to address in a clinical trial protocol and related documents*. (DOC 121 kb)

## Abbreviations

DON: Director of Nursing
GP: General practitioner
ICECAP-O: The Investigating Choice Experiments for the Preferences of Older People Capability index
MMSE: Mini Mental State Examination
PA: Physical activity
RCT: Randomised controlled trial
SMART: Specific, measurable, achievable, realistic and timely
SPIRIT: Standard Protocol Items Recommendations for Interventional Trials
TUG: Timed Up and Go test
WHO: World Health Organization

## References

[1] World Health Organization (2015). World report on ageing and health.

[2] Hallal PC, Andersen LB, Bull FC, Guthold R, Haskell W, Ekelund U (2012). Global physical activity levels: surveillance progress, pitfalls, and prospects. Lancet.

[3] Butler RN, Davis R, Lewis CB, Nelson ME, Strauss E (1998). Physical fitness: benefits of exercise for the older patient. 2. Geriatrics.

[4] World Health Organization (2011). Global Recommendations on Physical Activity for Health 65 Years and Above.

[5] Holthe T, Thorsen K, Josephsson S (2007). Occupational patterns of people with dementia in residential care: an ethnographic study. Scand J Occup Ther.

[6] den Ouden M, Bleijlevens MHC, Meijers JMM, Zwakhalen SMG, Braun SM, Tan FES (2015). Daily (in) activities of nursing home residents in their wards: an observation study. J Am Med Dir Assoc.

[7] Crocker T, Forster A, Young J, Brown L, Ozer S, Smith J. Physical rehabilitation for older people in long-term care. Cochrane Database Syst Rev. 2013;(2) CD004294 10.1002/14651858.CD004294.pub3 PMC11930398 23450551

[8] Chou CH, Hwang CL, Wu YT (2012). Effect of exercise on physical function, daily living activities and quality of life in the frail older adults: a meta-analysis. Arch Phys Med Rehabil.

[9] Forster A, Lambley R, Hardy J, Young J, Smith J, Green J, et al. Rehabilitation for older people in long-term care. Cochrane Database Syst Rev. 2009;(1):CD004294. 10.1002/14651858.CD004294.pub2 19160233

[10] Rydwik E, Frandin K, Akner G (2004). Effects of physical training on physical performance in institutionalised elderly patients (70+) with multiple diagnoses. Age Ageing.

[11] Faber MJ, Bosscher RJ, Chin A, Paw MJ, van Wieringen PC (2006). Effects of exercise programs on falls and mobility in frail and pre-frail older adults: a multicenter randomized controlled trial. Arch Phys Med Rehabil.

[12] Peri K, Kerse N, Robinson E, Parsons M, Parsons J, Latham N (2008). Does functionally based activity make a difference to health status and mobility? A randomised controlled trial in residential care facilities (the promoting independent living study; PILS). Age Ageing.

[13] Fiatarone M, O’Neill E, Doyle N, Clements K, Roberts S, Kehayias J (1993). The Boston FICSIT study: the effects of resistance training and nutritional supplementation on physical frailty in the oldest old. J Am Geriatr Soc.

[14] American Association of Retired People, American College of Sports Medicine, American Geriatric Society, Centers for Disease Control and Prevention, National Institute on Aging, Robert Wood Johnson Foundation (2001). National blueprint: increasing physical activity among adults age 50 and older.

[15] van der Bij AK, Laurant MG, Wensing M (2002). Effectiveness of physical activity interventions for older adults: a review. Am J Prev Med.

[16] Brittle N, Patel S, Wright C, Baral S, Versfeld P, Sackley C (2009). An exploratory cluster randomized controlled trial of group exercise on mobility and depression in care home residents. Clin Rehabil.

[17] Byrne SV (2016). Exercise beliefs in elderly nursing home residents: a cross-sectional, case control study [MSc thesis].

[18] Bandura A (2004). Health promotion by social cognitive means. Health Educ Behav.

[19] Anderson-Bill ES, Winett RA, Wojcik JR (2011). Social cognitive determinants of nutrition and physical activity among web-health users enrolling in an online intervention: the influence of social support, self-efficacy, outcome expectations, and self-regulation. J Med Internet Res.

[20] Noar SM, Chabota M, Zimmerman RS (2008). Applying health behavior theory to multiple behavior change: considerations and approaches. Prev Med.

[21] Chan AW, Tetzlaff JM, Altman DG, Laupacis A, Gøtzsche PC, Krleža-Jerić K (2013). SPIRIT 2013 statement: defining standard protocol items for clinical trials. Ann Intern Med.

[22] Calvert M, Kyte D, Mercieca-Bebber R, Slade A, Chan A, King MT (2018). Guidelines for inclusion of patient-reported outcomes in clinical trial protocols: the SPIRIT-PRO extension. JAMA.

[23] Podsiadlo D, Richardson S (1991). The timed “up & go” (a test of basic functional mobility for frail elderly persons). J Am Geriatric Soc.

[24] Nursing Homes Ireland, 2018. Directory. http://www.irishnursinghomes.eu. Accessed 15 Feb 2018.

[25] Black BS, Rabins PV, Sugarman J, Karlawish JH (2010). Seeking assent and respecting dissent in dementia research. Am J Geriatr Psychiatry.

[26] Clare L, Hindle JV, Jones IR, Thom JM, Nelis SM, Hounsome B (2012). The AgeWell study of behavior change to promote health and wellbeing in later life: study protocol for a randomized controlled trial. Trials.

[27] Garmendia M, Dangour A, Albala C, Eguiguren P, Allen E, Uauy R (2013). Adherence to a physical activity intervention among older adults in a post-transitional middle income country: a quantitative and qualitative analysis. J Nutr Health Aging.

[28] Garber CE, Blissmer B, Deschenes M, Franklin B, Lamonte M, Michael J (2011). Quantity and quality of exercise for developing and maintaining cardiorespiratory, musculoskeletal, and neuromotor fitness in apparently healthy adults: guidance for prescribing exercise. Med Sci Sports Exerc.

[29] McAuley E, Jerome GJ, Marquez DX, Elavsky S, Blissmer B (2003). Exercise self-efficacy in older adults: social, affective, and behavioral influences. Ann Behav Med.

[30] van der Ploeg HP, Merom D, Chau JY, Bittman M, Trost SG, Bauman AE (2010). Advances in population surveillance for physical activity and sedentary behavior: reliability and validity of time use surveys. Am J Epidemiol.

[31] Sylvia LG, Bernstein EE, Hubbard JL, Keating L, Anderson EJ (2014). A practical guide to measuring physical activity. J Acad Nutr Diet.

[32] Avery KNL, Williamson PR, Gamble C, O’Connell E, Metcalfe C, Davidson P (2017). Informing efficient randomised controlled trials: exploration of challenges in developing progression criteria for internal pilot studies. BMJ Open.

[33] Robinson TN, Wallace JI, Wu DS, Wiktor A, Pointer LF, Pfister SM (2011). Accumulated frailty characteristics predict postoperative discharge institutionalization in the geriatric patient. J Am Coll Surg.

[34] Laflamme GY, Rouleau DM, Leduc S, Beaumont E (2012). The timed up & go test is an early predictor of functional outcome after hemiarthroplasty for femoral neck fracture. J Bone Joint Surg Am.

[35] Shumway-Cook A, Brauer S, Woollacott M (2000). Predicting the probability for falls in community-dwelling older adults using the timed up & go test. Phys Ther.

[36] Nordin E, Rosendahl E, Lundin-Olsson L (2006). Timed “up & go” test: reliability in older people dependent in activities of daily living- focus on cognitive state. Phys Ther.

[37] van Iersel MB, Munneke M, Esselink RA, Benraad CE, Olde Rikkert MG (2008). Gait velocity and the timed-up-and-go test were sensitive to changes in mobility in frail elderly patients. J Clin Epidemiol.

[38] Hunt SM, McKenna SP, McEwen J, Williams J, Papp E (1981). The Nottingham health profile: subjective health status and medical consultations. Soc Sci Med.

[39] Baro E, Ferrer M, Va’zquez O, Miralles R, Pont A, Esperanza A (2006). Using the Nottingham health profile (NHP) among older adult inpatients with varying cognitive function. Qual Life Res.

[40] Bureau-Chalot F, Novella JL, Jolly D, Ankri J, Guillemin F, Blanchard F (2002). Feasibility, acceptability and internal consistency reliability of the Nottingham health profile in dementia patients. Gerontology.

[41] Parker SG, Peet SM, Jagger C, Farhan M, Castelden CM (1998). Measuring health status in older patients. The SF-36 in practice. Age Ageing.

[42] Kim H, Park I, Lee HJ, Lee O (2016). The reliability and validity of gait speed with different walking pace and distances against general health, physical function, and chronic disease in aged adults. J Exerc Nutr Biochem.

[43] Montero-Odasso M, Schapira M, Soriano ER, Varela M, Kaplan R, Camera LA (2005). Gait velocity as a single predictor of adverse events in healthy seniors aged 75 years and older. J Gerontol A Biol Sci Med Sci.

[44] Hollman JH, Childs KB, McNeil ML, Mueller AC, Quilter CM, Youdas JW (2010). Number of strides required for reliable measurements of pace, rhythm and variability parameters of gait during normal and dual task walking in older individuals. Gait Posture.

[45] Coast J, Flynn TN, Natarajan L, Sproston K, Lewis J, Louviere JJ (2008). Valuing the ICECAP capability index for older people. Soc Sci Med.

[46] Makai P, Brouwer WB, Koopmanschap MA, Nieboer AP (2012). Capabilities and quality of life in Dutch psycho-geriatric nursing homes: an exploratory study using a proxy version of the ICECAP-O. Qual Life Res.

[47] Makai P, Beckebans F, van Exel J, Brouwer WB (2014). Quality of life of nursing home residents with dementia: validation of the German version of the ICECAP-O. PLoS One.

[48] Herdman M, Gudex C, Lloyd A, Janssen M, Kind P, Parkin D (2011). Development and preliminary testing of the new five-level version of EQ-5D (EQ-5D-5L). Qual Life Res.

[49] Sandelowski M (2000). Whatever happened to qualitative description?. Res Nurs Health.

[50] Lewin S, Glenton C, Oxman AD (2009). Use of qualitative methods alongside randomised controlled trials of complex healthcare interventions: methodological study. BMJ.

[51] O’Cathain A, Hoddinott P, Lewin S, Thomas KJ, Young B, Adamson J (2015). Maximising the impact of qualitative research in feasibility studies for randomised controlled trials: guidance for researchers. Pilot Feasib Stud.

[52] Braun V, Clarke V (2006). Using thematic analysis in psychology. Qual Res Psychol.

[53] Grabowski DC, Aschbrenner KA, Rome VF, Bartels SJ (2010). Quality of mental health care for nursing home residents: a literature review. Med Care Res Rev.

[54] Zermansky AG, Alldred DP, Petty DR, Raynor DK (2007). Striving to recruit: the difficulties of conducting clinical research on elderly care home residents. J R Soc Med.

